# Between repulsion and attraction in serial biases: Replication of Chen and Bae (2024)

**DOI:** 10.1167/jov.25.8.13

**Published:** 2025-07-11

**Authors:** Juni B. Akselberg, Sara B. Cardona, Mikkel Dybvad, Lise Martine Karlstad, Malin Langemyr, Ingrid A. Mellingsæter-Jokic, Mats K. K. Moe, Amalie C. Solvang, Andrey Chetverikov

**Affiliations:** 1 Faculty of Psychology, University of Bergen, Bergen, Norway; 2 Department of Psychosocial Science, Faculty of Psychology, University of Bergen, Bergen, Norway

**Keywords:** serial dependence, bias, direction estimation, orientation, visual working memory, Stroop, attraction, repulsion, mouse tracking, stimulus history effect, perceptual decision-making, replication

## Abstract

What you see depends on what you have seen before, and commonly your perception is drawn toward the past. Such attractive biases, known as serial dependence, are well established for many visual features. Interestingly, Chen and Bae (2024, *Cognition*) recently reported a repulsive serial bias in a pointing direction estimation task that switched to an attractive one in the presence of a distracting task. At the same time, an analysis of response trajectories revealed a repulsive bias during response execution, irrespective of the condition. These surprising findings prompted us to attempt a replication. We confirmed the main findings of Chen and Bae. However, we also demonstrated that the overall direction and magnitude of the bias are relatively stable for a given observer, regardless of the condition. Furthermore, we found that already the very first moment in the response trajectory differed between conditions, showing a predominantly attractive bias for trials that ended with attraction. The results confirm the robustness of the original findings and pose a challenge for a simple Bayesian model of serial dependence, highlighting the need for computational models that can explain both attractive and repulsive biases.

## Introduction

Cognition is context-dependent. Whether estimating the colors of an object or deciding which bet to take, irrelevant contextual information strongly affects decisions. While textbooks describe many empirical demonstrations of such phenomena, their mechanisms remain elusive. For example, in perception and memory studies, estimates of stimulus features are often attracted to previously presented stimuli. This phenomenon, known as “serial dependence” (Fischer & Whitney, 2014), has been demonstrated for a wide range of features, from orientation and color to emotional expressions (see reviews in Cicchini, Mikellidou, & Burr, 2024; Manassi, Murai, & Whitney, 2023; Manassi & Whitney, 2024; Pascucci et al., 2023). This effect has been explained by the Bayesian models as a tendency of the brain to integrate previous and current inputs under the assumption of a “stable world,” which posits that objects tend to change little over time (Cicchini, Mikellidou, & Burr, 2018; Cicchini et al., 2024; Kalm & Norris, 2018; van Bergen & Jehee, 2019). But is this a sufficient explanation? Here, we aim to replicate the findings of a recent study demonstrating opposite, repulsive biases, which raises questions about the applicability of Bayesian integration as a mechanism explaining serial biases.

In a recent article, Chen and Bae (2024a, Experiments 1A/1B) described a particularly striking set of findings. In each trial, participants had to remember the direction in which a teardrop-shaped stimulus was pointing. After a delay, they reported the direction by rotating another teardrop shape to match the stimulus. Several surprising findings emerged from this study. First, the authors found that responses were biased away from the direction of a stimulus in the previous trial. This finding contrasts with the “standard” attractive serial dependence, although it aligns with results from other studies using the same or a similar paradigm (Bae & Luck, 2017, Bae & Luck, 2019; Bansal et al., 2023; Bliss, Sun, & D'Esposito, 2017; Chen & Bae, 2024b; Wang, Fang, & Whitney, 2024). They then demonstrated that this repulsion switches to attraction when an unrelated Stroop task (Stroop, 1935) is introduced during the delay period. They argued that this manipulation allows clarifying the role of working memory in serial biases: While most studies of serial dependence have a delay period before the report, the role of distraction during that period was not tested before. Finally, they analyzed the time course of the response, revealing that for most of the response execution period, observers were biased away from the previous stimulus. Only just before the decision was reported did this bias switch to attraction in the Stroop condition. This suggests that initially, the stimulus from the previous trial creates a repulsive influence that may switch to attraction later in the decision-making process. The combination of these three novel findings—the repulsive bias, its switch to an attractive one in the Stroop condition, and the time course of the bias—makes this study an enticing target for replication.

If robust, these findings would present a challenge for any model aiming to explain why and how serial biases occur. This is especially true for the Bayesian model, which can accommodate only attractive, but not repulsive, biases. Other, more descriptive models, such as those attributing repulsive effects to memory about the stimuli and attractive ones to memory about responses (Pascucci et al., 2019), would also struggle to explain the shift from one bias direction to another. That being said, some models (Chetverikov, 2023a; Fritsche, Spaak, & de Lange, 2020) may potentially account for this change in bias direction, and we will return to this question in the Discussion.

In addition, Chen and Bae (2024a) reported that response times in the pointing direction estimation task decrease linearly as a function of the similarity between the current and the previous item. This effect, they argued, supports the idea that “the serial bias is driven by the decision during the reporting” (Chen & Bae, 2024a, p. 2). While the debate about the processing stages that create serial dependence is not the focus of this article (see Manassi, Murai, & Whitney, 2023; Pascucci et al., 2023, for a discussion), the relationship between the response times and similarity is interesting from a theoretical point of view.

Here, we report a direct replication of Experiments 1A/1B by Chen and Bae (2024a) with a minor difference: We used a within-subject design instead of a between-subject design. Our main motivation for using a within-subject design was to ensure that the difference between the conditions cannot be explained by interindividual variability. Furthermore, within-subjects designs usually have a higher power compared to between-subject ones. In addition, this provided us with the opportunity to analyze the correlation between the biases across conditions, examining the role of individual difference more closely.

To preview our findings, we successfully replicated the original study, demonstrating repulsive response trajectories that ended with an attractive bias when an extra Stroop task was introduced but remained repulsive without it. However, we also show that these trajectories are likely to start with an attractive bias when they end with an attractive bias. In the Stroop condition, this leads to a positive starting bias on average, but this unexpected result must be treated with caution. Furthermore, biases appear to be stable across individuals, with most of the observers showing the same bias direction (attractive or repulsive) in both conditions.

## Methods

The study followed the design of Experiment 1A/B in Chen and Bae (2024a). The only minor difference was that we employed a within-subject design instead of a between-subject one, so each participant went through a condition with a Stroop task and the one without it (Experiments 1A and 1B in the original study, respectively) in a randomized order.

### Participants

Thirty-seven participants took part in the study. The data collection was anonymous, and no personal data were recorded. Most of the participants were recruited from the student pool at the University of Bergen and took part in the study in exchange for course credits. The rest of the participants were recruited through word of mouth and compensated with a gift card at the rate of 200 NOK (approx. 17 Euro) per hour. Four participants were excluded from analyses: three because they did not follow instructions for the Stroop task and one because they had very poor accuracy in the direction estimation task. Informed consent was given electronically at the beginning of the experiment.

### Design

All of the participants took part in two conditions corresponding to Experiments 1A (Stroop condition) and 1B (No Stroop condition) of Chen and Bae (2024a) in randomized order in two separate sessions on separate days. Sixteen and 17 participants started with No Stroop and Stroop conditions, respectively. The time between each session varied from 1 day to 10 days.

### Apparatus, stimuli, and procedure

The study was built and conducted using PsychoPy 2023.2.3/2024.2.2 (Peirce et al., 2019). The stimuli were presented on ASUS VG248 LCD monitors (144 Hz, 1,920 × 1,080 pixels, 53 cm wide), with participants seated approximately 60 cm away.

**Figure 1.**
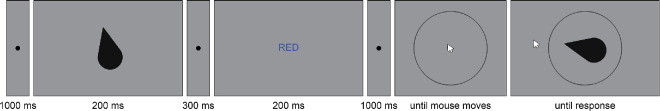
Task procedure. Participants had to remember the pointing direction of a teardrop-shaped stimulus. After a delay, they saw a Stroop stimulus—a word reading “RED,” “GREEN,” or “BLUE”—randomly colored in red, green, or blue. Participants were instructed to respond by pressing a keyboard key that matched the color of the word (Stroop condition) or to press the same key regardless of the stimulus (No Stroop condition) within a 1-s delay following the stimulus. They then saw a circle indicating that they should report the direction in which the teardrop shape was pointing using the mouse. Once the mouse started moving, the teardrop shape reappeared, following the direction of the mouse cursor. Participants indicated that they were done responding by pressing the left mouse button when the cursor was outside the response circle area.

In line with the original study, we utilized a dual-task paradigm with two conditions. Each trial began with a 1,000-ms presentation of a fixation point (black circle, 0.25° of visual angle [dva] in diameter; Figure 1), followed by a teardrop shape (2.5 dva) pointing in a random direction (chosen from a uniform distribution from 0° to 360°), displayed for 200 ms. Participants were required to remember the direction in which it was pointing. After a 300-ms delay, they saw a Stroop stimulus—a word RED, GREEN, or BLUE (letter height 0.57 dva), shown in red, green, or blue color (RGB primaries at full contrast) for 200 ms. In the Stroop condition, participants responded to the Stroop stimulus by pressing the left, down, or right arrow keys for the colors red, green, or blue, respectively. The keys on the keyboard were marked with corresponding colored stickers. In the No Stroop condition, participants always had to press the down arrow key, regardless of the stimulus. Following this, the fixation point reappeared for another 1 s. The response time was limited to 1.2 s in total in the Stroop task (including the Stroop stimulus presentation time). If the response time for the Stroop stimulus exceeded 1.2 s, the message SLOW (white letters, 0.57 dva in height) was presented for 500 ms.

Finally, a black circle (4.3 dva in diameter) and a mouse cursor appeared, indicating that participants needed to report the orientation of the teardrop shape they had seen earlier. Upon the first mouse movement, a teardrop-shaped probe, always oriented toward the mouse cursor, reappeared. Participants were required to align the probe as accurately as possible to match the orientation of the stimulus and press the left mouse button to submit their answer. To avoid accidental responses, the mouse cursor had to be outside the circle for the response to count. The cursor was repositioned to the center of the screen for each trial, and the response time was not limited.

Each condition consisted of an instruction page followed by 15 practice trials. In both conditions, participants were asked to complete a set of five blocks, each consisting of 96 trials, with self-paced breaks in between. They received feedback about their performance (the average error in direction estimation) during the breaks.

### Data preprocessing

#### Removing anisotropies in response errors

We employed an approach described in van Bergen, Ma, Pratte, and Jehee (2015) and further developed in the *circhelp* package (Chetverikov, 2023b) for use in R. This method is designed to mitigate attractive or repulsive biases toward cardinal directions, which are commonly observed in orientation and motion perception studies (Chetverikov & Jehee, 2023; Wei & Stocker, 2017) and can interact with serial biases (Bae, 2024). The goal is to enhance the signal-to-noise ratio in the data. While a detailed explanation is available in the package documentation, we will provide a brief summary here.

For each participant in our data sets, we generated two sets of bins. These bins divide the trials into groups centered on cardinal and oblique directions, as either repulsion or attraction to cardinals can occur. Repulsion from cardinals may cause a discontinuity in the average error relative to the pointing direction. To address this, we split the data into bins centered on oblique orientations. Conversely, attraction to cardinals (or repulsion from obliques) might be better addressed by grouping data around cardinal directions.

For each set of bins (i.e., those centered on cardinals and those centered on obliques), we fitted a fourth-degree polynomial to the responses within each bin, using the distance to the bin center as the independent variable and the response error as the dependent variable. We further allowed the width of the response distribution to vary as a function of distance to the nearest cardinal direction. Responses outside the ±3 × *SD* range were excluded from the fitting process but added back afterward. The model with the best-fitting set of polynomials was then chosen to represent the biases for a given participant. The mean predicted error was subsequently removed from each response, and responses outside the predicted ±3 × SE range were considered outliers and excluded from further analyses.

#### Estimating serial biases

We estimated serial biases as the asymmetry in response probability density toward or away from previous stimuli using the *density_asymmetry* function in the *circhelp* package in R (Chetverikov, 2023b). This approach is not sensitive to the magnitude of an error. Instead, it measures how likely it is to observe a shift in response to the previous stimulus. This makes it more robust and less sensitive to the differences in similarity between the items. For instance, when two items differ by 5°, the bias measured as mean error can theoretically be only up to 5°. However, when they differ by 40°, the bias can also go up to 40°, making the magnitude of bias difficult to compare.

The details of the approach are provided in the package documentation. Briefly, the sign of response errors is adjusted so that positive errors correspond to errors toward previous stimuli, while negative errors correspond to a bias away from them. Then, a probability density is estimated for errors in each condition (e.g., Stroop or No Stroop), and its asymmetry is computed by subtracting the probability density of negative errors from that of positive errors. The asymmetry is then normalized by the sum of the densities for positive and negative errors and multiplied by 100 to express it as a percentage. A 100% bias indicates that all errors were made toward previous stimuli, while −100% indicates that all errors were made away from previous stimuli. In the case of continuous variables, such as the dissimilarity between the previous and the current stimulus, a rolling Gaussian kernel is used to obtain a continuous estimate of the asymmetry.

For the sake of completeness and to match the analyses in the original Chen and Bae (2024a) article, we also present the results using the mean biases in Supplementary Figure S2. Confidence intervals were estimated using the *superb* package in R, which implements the necessary adjustments for repeated-measures designs (Cousineau, Goulet, & Harding, 2021).

## Results

### Stroop task performance

We first analyzed the performance in the Stroop task to ensure that participants paid attention to it and that it had the intended distracting effect. As expected, performance was significantly better in the Stroop condition when the color and the word were congruent, in terms of both response times (congruent vs. incongruent: *M* = 0.62 [0.60, 0.65] vs. *M* = 0.66 [0.63, 0.68], *t*(32.0) = 6.25, *p* < 0.001; here and later we report 95% confidence intervals in square brackets) and accuracy (*M* = 92.67 [90.41, 94.73] vs. *M* = 91.20 [88.49, 93.55], *t*(32.0) = −2.59, *p* = 0.014; see also Supplementary Figure S1). In the No Stroop condition, trial congruence did not affect response times (*M* = 0.38 [0.31, 0.45] vs. *M* = 0.38 [0.31, 0.45], *t*(31.0) = 0.28, *p* = 0.779) or accuracy (*M* = 34.44 [31.53, 38.78] vs. *M* = 30.85 [28.51, 32.53], *t*(31.0) = −1.22, *p* = 0.231), indicating that participants ignored the Stroop stimulus in this condition as intended. Note that the difference in response times between the Stroop and No Stroop conditions did not affect the delay between the orientation stimuli and responses, as the response interval duration in the Stroop task was fixed. Additionally, there were no significant differences in the number of trials with responses that were not given in time (Stroop vs. No Stroop: *M* = 0.03 [0.02, 0.05] vs. *M* = 0.04 [0.02, 0.06], *t*(32.0) = −0.58, *p* = 0.565), suggesting that the presence of “too slow” feedback cannot explain the potential differences between the conditions.

### Performance in the direction estimation task

We first looked at the average error in observers’ responses. As expected, the performance was worse when the observers had to engage in the Stroop task, again confirming that the Stroop task interfered with the main task (*M* = 8.82 [7.86, 9.84] vs. *M* = 7.04 [6.38, 7.87], *t*(32.0) = 4.15, *p* < 0.001, *d* = 0.72). Observers also responded faster in the Stroop compared to the No Stroop condition (*M* = 0.88 [0.82, 0.95] vs. *M* = 0.96 [0.89, 1.04], *t*(32.0) = −3.35, *p* = 0.002, *d* = 0.58), potentially reflecting differences in the precision of direction representations.

**Figure 2.**
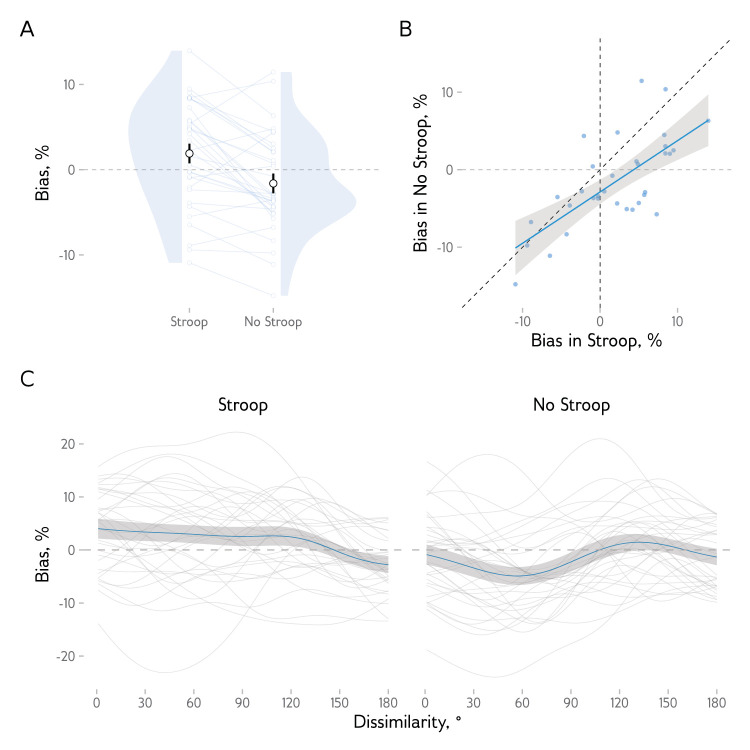
Serial biases in responses. (**A**) Observers show positive (attractive) biases in the Stroop condition and negative (repulsive) biases in the No Stroop condition. Large dots and bars show means and 95% within-subject confidence intervals for each condition. Small dots show the data from individual subjects, with lines connecting the results from the same observer. Gray regions show the probability density of observers’ mean biases. (**B**) Biases are strongly correlated between conditions. Each dot shows the data for a single participant. The solid line shows the fitted linear regression prediction, with the 95% confidence interval as the shaded region. (**С**) Response bias as a function of similarity between the current and the previous trial. Thin lines show the data from individual participants. Thick lines and shaded regions show the average data and the associated 95% confidence intervals.

Do observers show repulsive biases, and do these biases switch to attraction when they engage in an additional task? Turning to the main question of our analyses, we found that the response bias was repulsive in the case of the No Stroop condition (*M* = −1.61 [–2.76, −0.45]) and attractive in the case of the Stroop condition (*M* = 1.90 [0.75, 3.06]), with a significant difference between the two (*t*(32.0) = 4.39, *p* < 0.001). An additional analysis including the order of conditions showed no significant order effects, *F*(1, 31) = 0.54, *p* = 0.468, η^2^_G_ = .01, or the interaction between the order and condition, *F*(1, 31) = 1.01, *p* = 0.323, η^2^_G_ < .01. Interestingly, the average bias for each participant was strongly correlated between conditions, *r*(31) = 0.70, *p* < 0.001 (Figure 2B), suggesting common mechanisms for biases in the two conditions.

We then analyzed the magnitude of the bias as a function of dissimilarity between the stimuli direction on the current and previous trials. As shown in Figure 2C, the observers were biased toward the previous stimuli in the Stroop condition for a wide range of dissimilarity values (Student's *t*-tests at individual dissimilarity steps were significant at the *p* < 0.05 level from 1° to 35°). In contrast, in the No Stroop condition, the bias was mostly repulsive (*t*-tests significant from 26° to 87°).

In a control analysis, we confirmed that the attractive biases are not caused by the swap errors, that is, that observers do not simply report the previous target. We found instead that the biases stem predominantly from small errors irrespective of the relative direction of the previous target, which is inconsistent with the swap errors explanation (Supplementary Figure S3).

### Response trajectories

**Figure 3.**
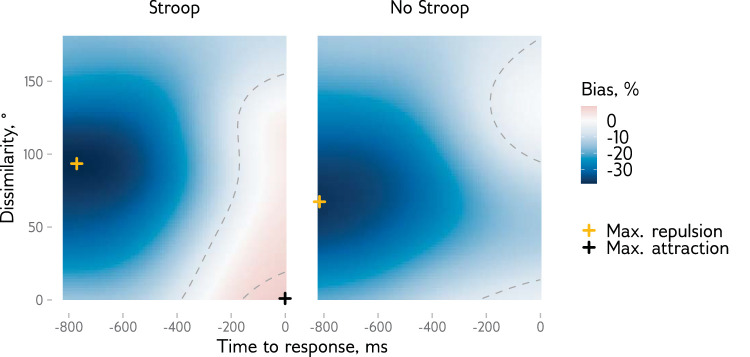
Within-trial response bias as a function of time before the final response and dissimilarity between the current and the previous stimuli. Colors show the bias direction. Dashed lines show the boundaries of significant (based on a *t*-test *p* < 0.05) clusters of repulsive and attractive biases, with crosses indicating the points with strongest biases. Note that for the No Stroop condition, only the repulsive bias cluster is present.

Following Chen and Bae (2024a), we further analyzed how the response trajectories develop over time (see Supplementary Figure S4 for example response trajectories). To do so, we estimated bias as a function of time to response (with a smoothing Gaussian kernel, *SD* = 120 ms) and dissimilarity (Gaussian kernel, *SD* = 20°) on a 90 × 90 points grid. We included only the time points (frames) where each observer had at least 20 trials, resulting in a selection of time points up until 817 ms before the response. We then tested for biases at each point at the time—dissimilarity space using a *t-*test with a *p* < 0.05 threshold. The results show that the bias was repulsive in both conditions and only later became attractive in the Stroop condition (Figure 3). The strongest repulsion was observed at −771 ms and 94° (*t*(32) = −15.97, *p* < 0.001) for the Stroop condition and at −817 ms and 67° (*t*(32) = −17.36, *p* < 0.001) for the No Stroop condition. Significant attraction was observed only for the Stroop condition, with the strongest effect at −1 ms and 1° (*t*(32) = 2.71, *p* = 0.011). In sum, responses in both conditions indicated repulsion during response execution, peaking at intermediate dissimilarity between the current and previous items that later switched to attraction in the Stroop condition, peaking at low dissimilarity.

### Biases at the start of the response period

We then asked whether the repulsive response trajectories actually indicate a switch in the bias sign from repulsion to attraction in the Stroop condition during the response execution. The previous analysis followed the approach taken by Chen and Bae (2024a) by looking at times leading to response. However, observers decide themselves how much time they want to spend responding, which means that in this response-locked analysis, the beginning of a trial is missing for long trials (above 817 ms) and varies in time for other trials. This makes it difficult to infer the initial direction of a bias for a given condition.

We thus analyzed the bias at the first frame as a function of the bias at the final frame for a given trial. Even though the mouse cursor was hidden before the trial start and repositioned at the center of the display when a trial began, observers could initiate the mouse movement in advance. As mouse position is polled independently of what is shown on a screen (and with a higher frequency), the first recorded coordinate does not necessarily have to be at zero. We reasoned that if observers’ representations are initially repulsed by or attracted to the previous item, we would observe this effect at the start of a response. If the bias switches to attraction mid-response, then the sign of the final response should not matter; all responses would begin with repulsion, or no bias would be observed if the noise at the initial time points is too high.

**Figure 4.**
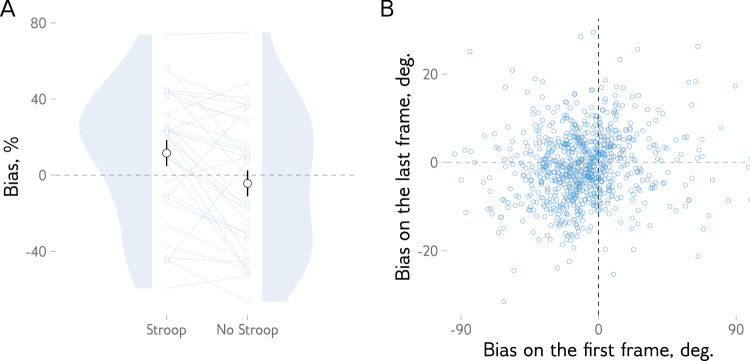
Response bias at the start of a response depends on the condition and predicts the final bias. (**A**) Observers are more likely to begin with an attractive bias in the Stroop condition. Large dots and bars represent the means and 95% within-subject confidence intervals for each condition. Small dots show the data from individual subjects, with lines connecting the results from the same observer. Gray regions show the probability density of observers’ mean biases. (**B**) Data from an example observer show the correlation between biases at the first and last time points within a trial. Each dot is a single response. Responses that end with an attractive bias are more likely to start with an attractive bias, and the same is true for responses that end with a repulsive bias.

We found instead that the initial response biases were more likely to be attractive than repulsive when the trial ended with an attractive bias in the Stroop condition (*M* = 0.22 [0.11, 0.32], *t*(32.0) = 3.91, *p* < 0.001 with sign coded as +1 for attractive and −1 for repulsive). The opposite was true at the tendency level in the No Stroop condition when the trial ended with a repulsive bias (*M* = −0.12 [–0.24, 0.01], *t*(32.0) = −1.86, *p* = 0.072). No significant differences were found when the trial ended with a repulsive bias in the Stroop condition (*M* = 0.01 [–0.10, 0.13], *t*(32.0) = 0.25, *p* = 0.805) or when it ended with an attractive bias in the No Stroop condition (*M* = 0.04 [–0.09, 0.16], *t*(32.0) = 0.61, *p* = 0.545). In other words, the condition had an effect already during the first moment in the response trajectory, with a significantly higher chance of a positive bias in the Stroop condition (*t*(32.0) = 3.39, *p* = 0.002, Figure 4A; see also Supplementary Figure S5). Furthermore, a generalized mixed-model analysis showed that the sign of the bias at the first moment predicted the sign of the bias at the last moment in response (see example participant in Figure 4B; *z* = 9.99, *p* < 0.001; random slope and intercept effects are included in the model). The attractive bias during the response initiation in the trials ending with attractive bias was short-lived: Already by the second frame, most of these trials (42.97%) showed repulsive bias. Together, these results suggest that the extra task affects response biases already during the response initiation.

### Response times as a function of similarity between the items

**Figure 5.**
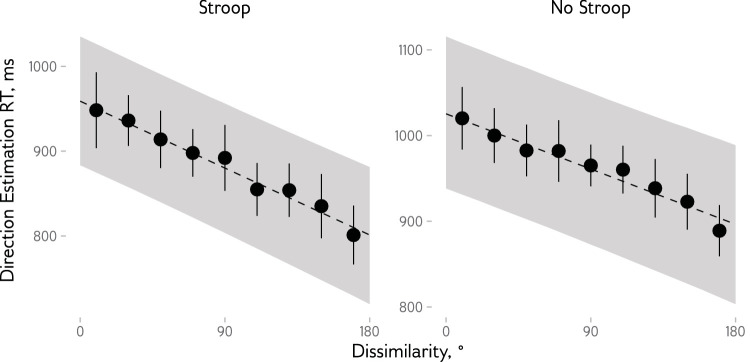
Response time (RT) in the pointing direction estimation task as a function of the dissimilarity between the current and previous targets and condition. Points show the mean RT for dissimilarity binned in 20° bins, with 95% confidence intervals shown with vertical bars. Dashed lines show the average predicted by a Bayesian hierarchical regression model, with shaded regions indicating 95% quantile-based credible intervals.

Finally, we analyzed how the similarity between the items in the current and previous trials affects response times in the direction estimation task. We used a Bayesian hierarchical regression model with participants included as a grouping factor and fixed and random effects for dissimilarity and condition. The model showed that response times decreased as dissimilarity between the current and previous targets increased (*b* = −0.88, 95% highest posterior density interval [HPDI] = [–1.07, −0.68] ms per degree of dissimilarity; Figure 5). There was also a main effect of condition (*b* = 66.42, 95% HPDI = [17.20, 116.09]) but no interaction effect (*b* = 0.16, 95% HPDI = [–0.03, 0.33]).

## Discussion

We aimed to replicate three surprising findings recently reported by Chen and Bae (2024a): (1) the overall repulsive serial bias in a pointing direction estimation task that (2) switches to attraction when an extra task is introduced, despite (3) the response trajectory showing strong repulsion in the course of decision-making, irrespective of the extra task present. Our results show support for all three, despite minor differences in the design and analytic approach, suggesting the robustness of the original findings. However, we also (4) find strong and stable individual differences in the bias direction and magnitude. We further show (5) that response trajectories are more likely to start with an attractive bias in the Stroop condition and (6) that the initial bias at the start of the response predicts the final response bias. Finally, we also replicated (7) the relationship between response times and the similarity between the current and the previous target.

### What drives repulsive biases in this task?

Unlike many other tasks showing attractive serial biases in perceptual decision-making (Cicchini et al., 2024; Manassi, Murai, & Whitney, 2023; Pascucci et al., 2023), this task shows repulsive serial biases. This is not the only known case of repulsive serial biases, but the other studies show repulsion from items that are unattended or are not reported (Ceylan & Pascucci, 2023; Fischer & Whitney, 2014; Pascucci et al., 2019; Pascucci & Plomp, 2021). Here, in contrast, the repulsive bias is shown in a stimulus-report paradigm, similar to “standard” serial dependence conditions. While this repulsion was shown using the same task in several other studies by Bae and colleagues (Bae & Luck, 2017, Bae & Luck, 2019; Bansal et al., 2023; Chen & Bae, 2024b), to the best of our knowledge, there has not been a discussion of what causes this discrepancy with other paradigms.

Repulsive serial biases could hypothetically reflect a combination of two methodological aspects of the task: its reliance on spatial memory and a short delay between the stimulus and the response. While we describe the remembered visual feature as “pointing direction” (and Chen & Bae [2024a] describe it as “orientation”), it can also be treated as a spatial memory task because participants can remember the point where the shape is pointing. Serial biases are usually also attractive for spatial memory tasks (Barbosa et al., 2020; Luo, Zhang, & Luo, 2022; Manassi, Liberman, Kosovicheva, Zhang, & Whitney, 2018; Stein et al., 2020), yet Bliss et al. (2017) and Stein et al. (2020) reported that serial biases for locations are repulsive with immediate response but become attractive with longer delays. Wang et al. (2024) further recently found repulsive biases in a task where observers had to quickly move the cursor toward a target location. Similarly, Bansal et al. (2023) used the same task as we employed here with varying delays and found a repulsive bias with zero delay and attractive biases with longer delays. Note that this effect of delay might be specific to this particular type of task. Other tasks, such as orientation reports (Fritsche, Mostert, & de Lange, 2017), found attraction also with very short (300-ms) delays, and the seminal study by Fischer and Whitney (2014) used 1,250-ms delays comparable to the present study. The only exception with attractive serial dependence for spatial tasks without a response delay comes from Manassi et al. (2018), in which particularly low-contrast stimuli were used. The same study did not find significant repulsion or attraction with medium-contrast stimuli. Finally, while preparing this article, we have also become aware of another preprint by Chen and Bae (2024b) that showed attractive biases in the same task employed here but the spatial uncertainty was introduced (i.e., stimuli appeared in different locations). Overall, however, the existing studies seem to suggest that a short response delay in spatial memory tasks might result in repulsive biases.

Interestingly, we observe considerable individual differences in the direction of biases. While on average, the bias was repulsive in the No Stroop condition and attractive in the Stroop condition, a significant number of observers exhibited attractive (*n* = 11) or repulsive (*n* = 11) biases in both conditions. This is more than the number of observers for whom the biases actually switched sign (*n* = 9). Overall, the magnitude and direction of the bias were significantly correlated between the conditions. All this suggests that the bias direction should be treated more as a continuum rather than strictly attractive or repulsive. At the same time, ∼60% of participants in the No Stroop condition did show a repulsive bias, in contrast to ∼23% of observers showing repulsion in typical orientation tasks (Manassi & Whitney, 2024). So, we argue that the question of why the biases in this type of task are repulsive is still puzzling, even though individual differences are present.

Our findings suggest that observers’ responses are already biased at the start of the response. Responses that end with an attractive bias are more likely to begin with an attractive bias, while those that end with a repulsive bias are more likely to start with a repulsive bias. Furthermore, the bias in the Stroop condition was significantly more positive than in the No Stroop condition during this first frame. This occurs despite the predominantly repulsive biases observed during the response that are seen irrespective of the condition and the final response bias. Note that Chen and Bae (2024a, Supplementary Figure S7) report mostly repulsive biases when the response was initiated (cf. our Supplementary Figure S5). In a different study using the same task but with the spatial uncertainty (i.e., varying location of the stimulus) instead of the Stroop task, Chen and Bae (2024b) also report repulsive initial biases. Interestingly, in the most recent version of their preprint, Chen and Bae (2024b) also notice the difference in the initial bias between conditions and the correlation between the initial and the final bias, but they analyze it in terms of the magnitude of the bias rather than the consistency in the sign. The difference in the results between the current study and the work of Chen and Bae (2024a) is unlikely to be driven by differences in analytic approaches, as we observed the same results using the raw data and the mean error as a measure of bias (Supplementary Figure S6). Although the initial attractive bias in our data is evident only in the first frame, its correlation with the final bias and the differences between the conditions suggest that it is not an artifact. We do not have a clear explanation of what prompts the divergence with the original study in terms of the sign of the initial bias but note that the angle of the trajectory is very noisy at the start of the response (i.e., given that it is close to the point of origin, one pixel shift in cartesian coordinates would mean a large change in angle). Furthermore, technical aspects (the type of mouse used or the way its coordinates are polled by the experimental software) might potentially be important in determining the initial bias.

These initial bias results complicate the task of pinpointing the origins of the repulsive and attractive biases. However, while Chen and Bae (2024a) suggested that “response trajectories started from the repulsion,” our additional analyses indicate that this is not the case. Instead, both repulsion and attraction in the response bias trajectories appear to occur both before and during the response.

### Repulsive and attractive serial biases from the normative perspective

What do the results mean for the theory of serial dependence? While “there is no shortage of modelling work on serial dependence” (Manassi & Whitney, 2024, p. 362), we focus here on normative models that explain serial dependence as a by-product of behavior that is optimal for a given task. This approach is beneficial because the constraints imposed by optimality principles reduce the flexibility of such models and allow for an explanation of behavior, in contrast to descriptive models that may provide only a description without an explanation (Geurts et al., 2018).

Most prominent among these are Bayesian models suggesting that observers integrate information from previous stimuli with current ones (Cicchini et al., 2018; Cicchini et al., 2024; Fritsche et al., 2020; Kalm & Norris, 2018; van Bergen & Jehee, 2019). This behavior is optimal under the ecologically valid assumption that most objects change relatively little over time. The repulsive biases pose a challenge for a purely Bayesian model, as information integration can only lead to attractive biases. However, Bayesian models can explain the strengthening of the attractive bias in the Stroop condition. In this framework, introducing an additional task might be treated as extra noise added to the computations. For the Bayesian model, this would mean a stronger attraction to previous items, assuming that the extra noise affects the representation of the current item (“likelihood” in Bayesian terms) more than the memory of the previous one (“prior”). In summary, the Bayesian model can account for the differences between the Stroop and No Stroop conditions, even though it cannot explain why the biases are repulsive in the No Stroop condition or the observed repulsion in the analysis of response trajectories.

The repulsion biases and their transition to attraction can be explained by assuming that repulsive and attractive biases coexist independently. In particular, Fritsche et al. (2020) combined a Bayesian model with separate changes related to the sensory encoding of visual stimuli using the “efficient coding” approach (e.g., Wei & Stocker, 2015). According to this model, the visual system reallocates sensory processing capacities to accurately represent new stimuli that match previous inputs. This can lead to repulsive biases when new stimuli do not match what was seen before. In the context of the current study, the efficient coding aspect may explain why the observed response trajectories are repulsive and why an overall repulsive bias is noted in the absence of a secondary task.

Notably, this efficient Bayesian observer model assumes that the efficient coding component is somewhat separate from the Bayesian component. This separation theoretically allows the model to account for any bias pattern, but it simultaneously reduces its predictive power. If the model is made more stringent by reintroducing the link between its components, its ability to explain the data is significantly diminished (Fritsche et al., 2020). Additionally, while efficient coding is meant to occur during stimulus encoding, as indicated by its name, the results here suggest that repulsion also occurs during the actual report. It is possible that stimuli are initially encoded with even stronger repulsion, and what is observed in the response trajectories analysis is merely the Bayesian component exerting its influence. However, we have also observed that responses leading to a positive bias are likely to start with a positive bias, raising questions about this explanation.

Wang et al. (2024) suggested that repulsive serial biases are linked particularly to efficient coding of a motor plan. While this study does not allow for disentangling motor planning from perception, it is unclear how this explanation would explain the difference between the conditions, as the motor component of the task stays intact. Furthermore, this proposal disagrees with previous studies suggesting that it is actually the stimulus that creates repulsion while responses create attraction (e.g., Sadil, Cowell, & Huber, 2024; Sheehan & Serences, 2023).

In summary, the efficient Bayesian observer model can account for both repulsive and attractive biases, but the loose connection between the efficient and Bayesian components undermines its appeal from a normative perspective, and the data appear to contradict the notion of repulsion occurring only during encoding.

Alternatively, the repulsive biases and the switch to attraction can be explained by the Demixing Model (Chetverikov, 2023a). In brief, the model suggests that stimuli existing close in time or space (here, the stimuli from the current and previous trials) create a mixture of neural signals that need to be separated, or “demixed,” to estimate the properties of the stimuli. The model describes an optimal solution for this problem and shows that this solution results in biased estimates. Crucially, the direction and magnitude of the biases depend on the amount of noise in the signals and the distribution of that noise across response-related (e.g., pointing direction) and stimulus-identifying (e.g., time) dimensions.

The noise in the stimulus-identifying dimension might be particularly relevant for understanding the results described above, namely, the repulsion and its switch to attraction with increased delays or when an extra task is introduced. The model predicts that as the overlap across this dimension increases, the biases become more attractive. Importantly, this attraction is not driven by swap errors, stemming instead from partial attribution of sensory signals coming from one stimulus to another. With short report delays, the current and previous stimuli are easily distinguishable. As the time before the report increases, the amount of noise due to decay or interference in memory increases, leading to a decrease in the signal-to-noise ratio for the signals identifying which stimulus was actually the last one. This could lead to a switch from repulsion to attraction with increasing response delays.

In a similar fashion, the Demixing Model can also explain the stronger attraction in the Stroop condition compared to the No Stroop condition. Additional noise introduced by the Stroop task could lead to higher confusability of the two items on the identifying dimension, resulting in a stronger attractive bias. Finally, the same explanation could be applied to the recent findings of a shift toward attraction when spatial uncertainty is introduced (Chen & Bae, 2024b). In sum, this model would ascribe the repulsion to observers’ ability to discriminate the signals related to the current and previous stimuli, which might deteriorate in the presence of delays or an extra task, leading to a shift toward attraction.

The Demixing Model also predicts that the relative amount of noise for the two stimuli on the response dimension could affect the direction of the biases. In particular, a less noisy item is predicted to be attracted to a noisier one, while the latter would be repelled from the former (a prediction that contrasts with a Bayesian model typically predicting an opposite pattern). To illustrate, if you see two overlapping patterns of colored tiles, with one of them in mostly blue colors and another with blue to green spectra, the blue tiles are likely to be seen as a part of the blueish pattern, even though some of them originate from the blue-green one. Then this blue-green pattern will be seen as greener than it is—a repulsion away from the blue color. Chetverikov (2023a) speculated that this could explain attractive serial dependence effects, as a more recent item is likely to be a less noisy source of neural signals than the one further back in memory. This prediction, however, is relatively difficult to test. First, the previous items are already encoded when the new one appears, and this dynamic is unaccounted for in the model. This also makes the model, in its current form, inapplicable for explaining the biases in response trajectories (although it could potentially be extended to predict the decision dynamics). Second, memory about previous reports further complicates matters. This makes estimating and testing the effects of relative noise levels difficult in the case of serial dependence.

In summary, the dynamics of serial biases in the results reported by Chen and Bae (2024a) and replicated here can be explained by both the efficient Bayesian observer model, which assumes independent attractive and repulsive biases, and the Demixing Model, which views them as a result of a single process. However, explaining existing results is easy. A strong test of these models requires hypotheses about not yet observed data. Ideally, one would predict when repulsive or attractive biases should emerge based on model parameters, such as stimulus noise levels or between-item similarity. This underscores the need for further theoretical advancement in the field.

### What does the relationship between response times and similarity mean?

While neither the Bayesian model nor the Demixing Model tackles response times in serial decisions directly, both can be linked to response times through uncertainty or noise in the underlying representations. The Bayesian model can be linked to response times by observing that the more uncertainty there is in the observer's representation of a stimulus, the more difficult it is to distinguish between this and similar stimuli, thus leading to longer decisions. More formally, the Bayesian observer's uncertainty can be shown to be inversely related to drift rates in the drift-diffusion models of response times for simple forced-choice decisions (Bitzer, Park, Blankenburg, & Kiebel, 2014), although we are unaware of a formal analysis of this relationship for continuous decisions. For the Demixing Model, on the other hand, response times are related to the difficulty of separating the signals. For example, the expectation-maximization algorithm used to derive the optimal solutions for a given set of sensory observations in Chetverikov (2023a) will take longer time to converge when stimuli are more similar to each other. This is because there is more ambiguity about which signal is caused by which stimulus, requiring the algorithm to take smaller steps and converge more slowly. Both models are thus capable of explaining response times, even though usually, response times are not the main focus of analyses.

Interestingly, the predictions by the Bayesian model and the Demixing Model about response times as a function of similarity between the items will be different. The Bayesian model predicts that posterior uncertainty will follow an inverted U-shaped curve as a function of similarity. The lowest uncertainty—and hence the fastest responses—will be predicted when stimuli on the current and previous trials are identical. In contrast, the Demixing Model will predict a decline in response times as stimuli become more dissimilar, as the underlying signals are then easier to disentangle. The two models thus predict contrasting patterns of results.

Our data replicate previous results by Chen and Bae and shows that response times are slowest when stimuli are identical and decrease with a decrease in similarity. These results match the predictions of the Demixing Model but not the Bayesian model. Further studies might be needed to test the generality of this finding across different feature domains.

## Conclusions

In summary, we were able to replicate the main findings of Chen and Bae (2024a), which showed repulsive biases in the delayed pointing direction estimation task and a shift to attraction with the addition of an extra task during the delay period. We also observed stable individual differences in bias direction and magnitude and demonstrated that attraction and repulsion enact their influences both before and during the response. These surprising findings cannot be explained by a pure Bayesian model of serial biases; however, they can be accounted for, with some caveats, by the Bayesian model combined with another process, such as efficient coding (Fritsche et al., 2020), or by the Demixing Model (Chetverikov, 2023a), which treats both attraction and repulsion as resulting from a single process. These models can also potentially explain the differences in response times, but they require further development to account for dynamical changes in bias magnitude and direction evident from analysis of response trajectories. Further studies are needed to provide a more rigorous test of the models and explain the mechanisms of serial biases in visual memory and perception.

## Supplementary Material

Supplement 1
