# Supplementary material for: Between repulsion and attraction in serial biases: Replication of Chen and Bae (2024)
Source: J Vis. 2025 Jul 11;25(8):13. doi: 10.1167/jov.25.8.13 (PMC12266288; doi:10.1167/jov.25.8.13)
Supplement: Supplement 1 [file jovi-25-8-13_s001.pdf]

Supplementary figures

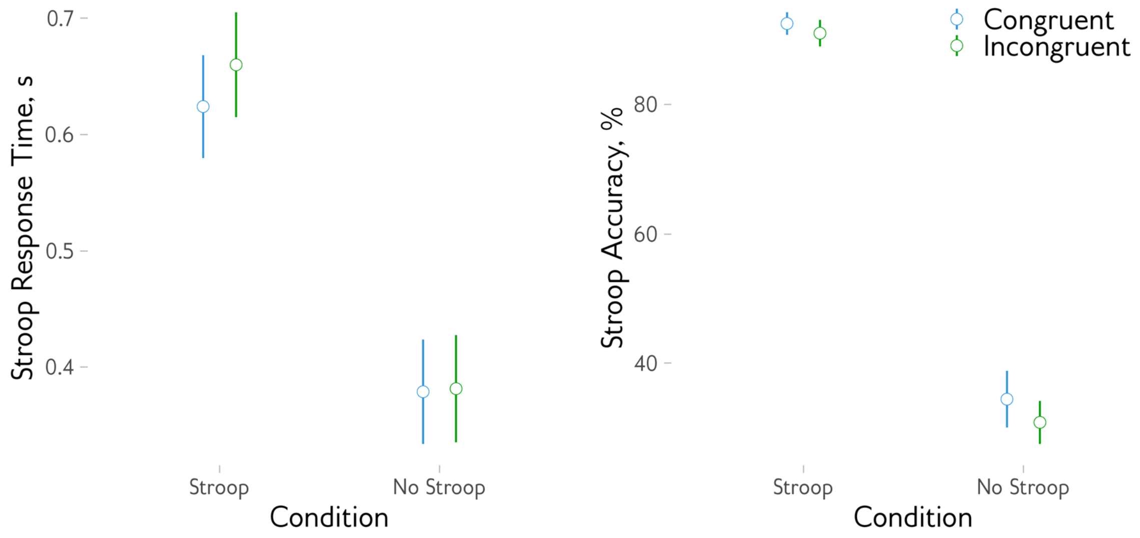

Figure S1: Response times and accuracy in the Stroop task. Points show group averages, bars show 95% confidence intervals.

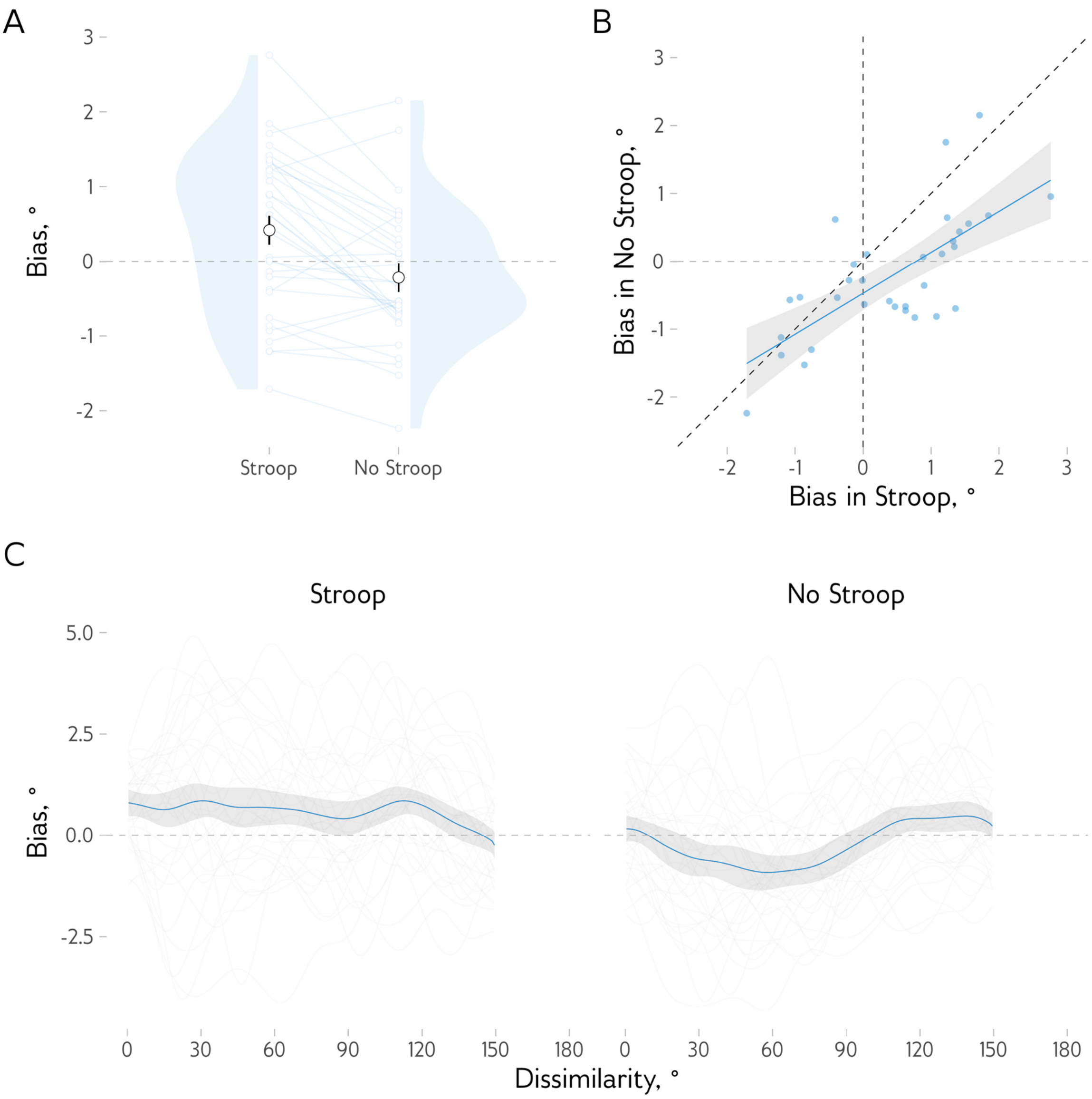

Figure S2: Bias estimates using mean error instead of asymmetry in probability densities. This analysis replicates the findings in Figure 2 using the same approach to measuring bias as used by Chen & Bae (2024a). **A** Mean bias. Large dots and bars show means and 95% within-subject confidence intervals for each condition. Small dots show the data from individual subjects, with lines connecting the results from the same observer. Gray regions show the probability density of observers' mean biases. **B** Biases are strongly ($r(31)$ = 0.71, $p < .001$) correlated between conditions. Each dot shows the data for a single participant. The solid line shows the fitted linear regression prediction, with the 95% confidence interval as the shaded region. **C** Response bias as a function of similarity between the current and the previous trial. Thin lines show the data from individual participants. Thick lines and shaded regions show the average data and the associated 95% confidence intervals.

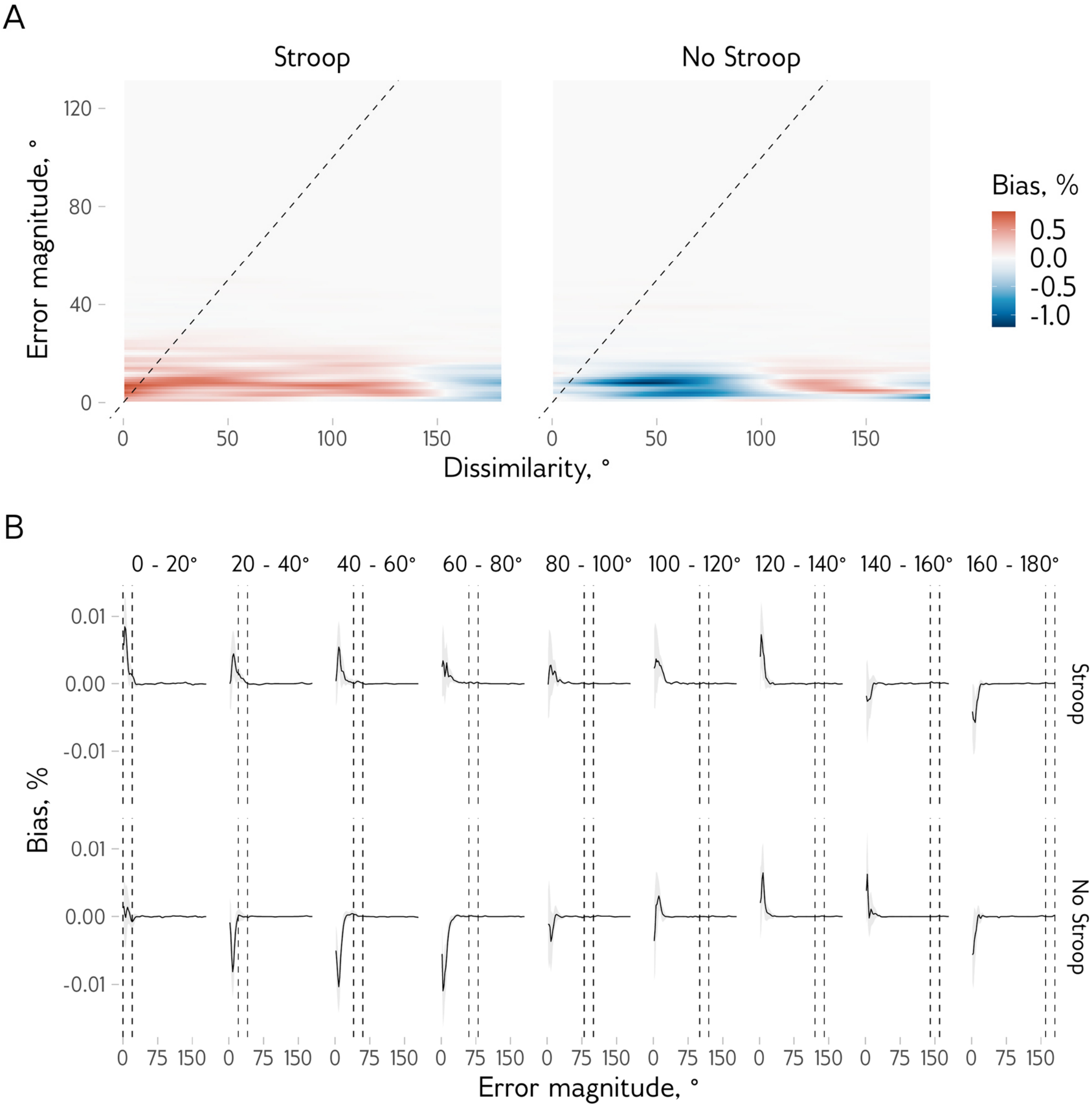

Figure S3: Bias for errors with different magnitudes. In this analysis, we asked what types of errors lead to observed biases. We were particularly interested in whether swap errors can explain the attractive bias observed in the Stroop condition. We analyzed the asymmetry of error probabilities in the same way as in the main text, but with consideration for error magnitude. **A**. Two-dimensional analysis of bias (asymmetry in error probabilities) as a function of error magnitude and dissimilarity between the current and previous stimuli. Colors indicate the direction of bias (red - attractive, blue - repulsive). The dashed line marks the hypothetical location of swap errors. If attractive biases were observed due to swap errors, we would expect to see a stronger bias around the dashed lines. However, we observe biases for relatively small errors, regardless of dissimilarity. **B**. Analysis of bias for bins of trials with different dissimilarities, as indicated in the facet labels. The dashed lines correspond to the regions where errors match the bin range. If attractive biases were due to swap errors, we would expect to see positive peaks between the dashed lines in each panel. However, consistent with the results in A, we observe biases for relatively small errors across most bins in the Stroop condition.

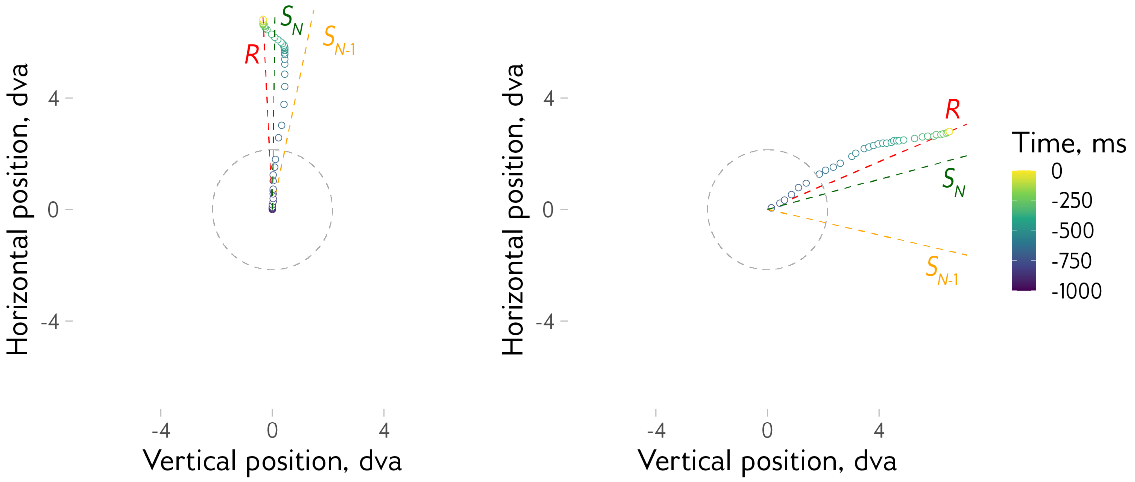

Figure S4: Response (mouse) trajectories for two example trials. S_N - the current stimulus angle, S_{N-1} - the previous stimulus angle, R - the response angle. Dashed circle shows the response boundary. Observers had to move the mouse cursor outside of this circle to respond. Dots show positions of the mouse cursor at individual frames with their color indicating time since the appearance of the response cue. Dashed lines show the hypothetical zero-bias trajectory matching the stimulus.

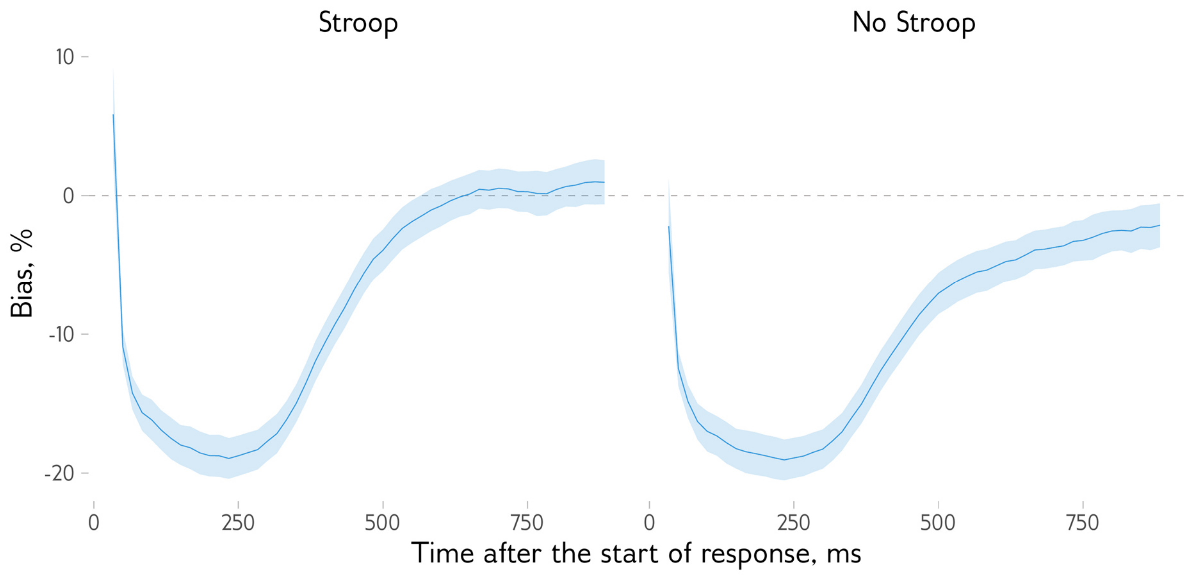

Figure S5: Within-trial response bias as a function of time after the response initiation. Lines indicate the mean bias, shaded regions indicate 95% confidence intervals. The data shows a positive bias in the very first time point of in the Stroop condition ($t(32.0) = 2.94$, $p = .006$) but not in No Stroop condition ($t(32.0) = 1.06$, $p = .296$; a comparison between conditions was also significant $t(32.0) = 2.15$, $p = .039$).

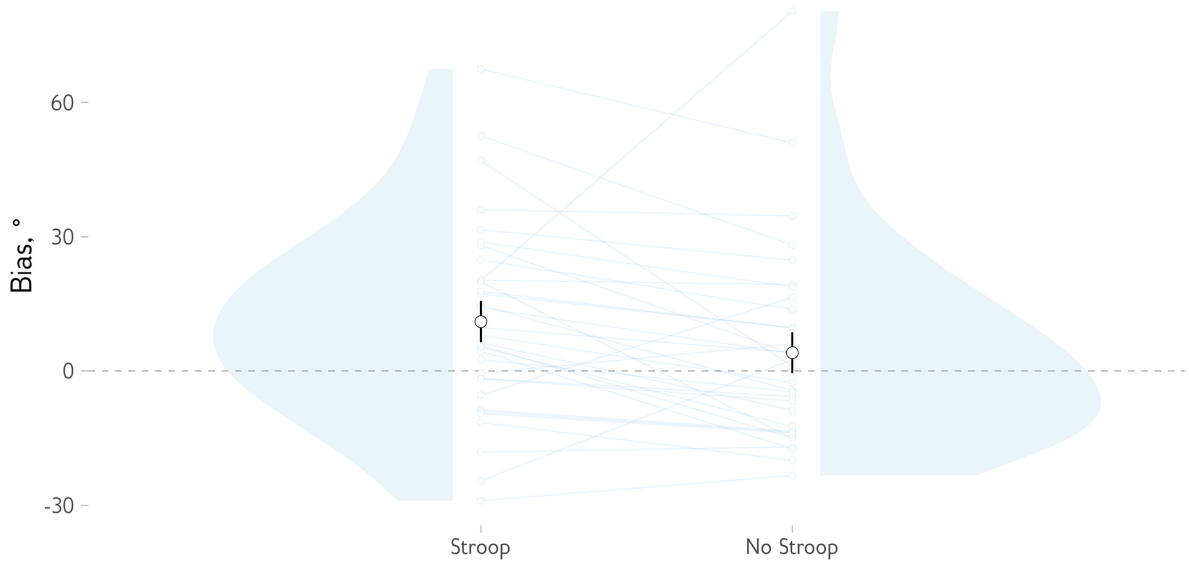

Figure S6: Analysis of the raw serial bias data on the first frame in the response trajectory. This analysis is done without any preprocessing (i.e., no removal of cardinal biases or outliers) and using the mean as measure of bias instead of density asymmetry. The results match Figure 4 with the Stroop condition showing significant attraction to the previous trial item. We also confirmed that the bias on the first frame predicts the final response bias when the raw data is analysed using a linear mixed model (B = 0.02, SE = 0.01, $t(7.64)$ = 2.83, p = .023).
